# A comprehensive dataset for sentiment and emotion classification from Bangladesh e-commerce reviews

**DOI:** 10.1016/j.dib.2024.110052

**Published:** 2024-01-14

**Authors:** Mohammad Rifat Ahmmad Rashid, Kazi Ferdous Hasan, Rakibul Hasan, Aritra Das, Mithila Sultana, Mahamudul Hasan

**Affiliations:** Department of Computer Science & Engineering, East West University Bangladesh, Jahurul Islam Ave, Dhaka 1212, Bangladesh

**Keywords:** Sentiment, Emotion, Manual annotation, Dataset, Multilabel, Multiclass, All categories review, Bangladesh

## Abstract

In the rapidly evolving domain of e-commerce, analyzing customer feedback through reviews is crucial, particularly for understanding and enhancing consumer experience in the Bangladeshi market. Our comprehensive dataset, derived from two Bangladeshi e-commerce platforms, Daraz and Pickaboo, features a diverse collection of reviews in both Bengali and English, covering a broad range of products. These reviews are not only rich in linguistic variety but also encapsulate a spectrum of emotions, some even conveyed through emojis, offering a deep dive into consumer sentiment. Expert annotators have meticulously examined and categorized each review, classifying emotions into five distinct types - Happiness, Sadness, Fear, Anger, and Love - and sentiments into Positive (Happiness, Love) and Negative (Sadness, Anger, Fear) categories. This level of detailed annotation enables precise assessments of customer emotions and preferences, which are essential for evaluating and improving existing product offerings. Moreover, the insights gleaned from this dataset are invaluable for guiding future product development and uncovering new opportunities in the dynamic Bangladeshi market. Ultimately, this dataset not only serves as a significant resource for sentiment analysis using natural language processing (NLP) techniques but also contributes valuable insights into the unique consumer behavior patterns in Bangladesh, enriching the NLP community's understanding of diverse market dynamics.

Specifications Table

**Table:** 

Subject	Machine Learning, Data Science, Computer Science, Statistical Analysis, and Data Mining
Specific Subject Area	This dataset, designed for advanced machine learning tasks within the Bangla language as part of Natural Language Processing, is essential for in-depth product performance analysis. It incorporates multiclass, multilabel sentiment data, annotated with five emotion labels - Sadness, Fear, Anger, Happiness, and Love - and two sentiment classes, Positive and Negative. Such detailed annotation enables precise assessments of customer emotions and preferences, crucial for evaluating and enhancing current product offerings. Furthermore, the insights derived from this dataset are invaluable for guiding future product development and exploring new opportunities in the Bangladeshi market.
Data Format	Annotated, Filtered, Raw, Analyzed
Type of Data	Text, Table
Data Collection	The dataset was meticulously compiled by extracting product reviews from Daraz and Pickaboo, two of the most prominent e-commerce platforms [10] in Bangladesh, using web scraping techniques. For this process, the Selenium automation framework was employed alongside a configured Chrome browser, ensuring efficient and accurate data collection. Each review in the dataset is richly detailed, containing several key attributes: Rating, Review text, Product Name, Product Category, Emotion, Sentiment, and the Data Source. These attributes provide a comprehensive view of each review, capturing not only the customer's emotional response (annotated with five emotions: Sadness, Fear, Anger, Happiness, and Love) but also the overall sentiment (Positive or Negative). Finally, all the data, initially saved in separate CSV files, were merged into a single CSV file to form a unified and extensive dataset.
Data Source Location	The reviews were collected at the East West University, Bangladesh, Primary Data Source (e-commerce platform): www.daraz.com.bd, www.pickaboo.com
Data accessibility	Repository Name: Mendeley Data Data identification number: 10.17632/rzjfg7t9kf.1 Direct URL to Repository: https://data.mendeley.com/datasets/rzjfg7t9kf/1

## 1 Value of the Data

• This dataset, encompassing a vast array of 78,130 product reviews from Daraz^1^ and Pickaboo^2^, two Bangladeshi e-commerce platforms, presents an in-depth view of consumer sentiment and emotion in the burgeoning Bangladeshi online market. The dataset's linguistic diversity, with reviews in both Bengali and English, makes it an ideal resource for analyzing and understanding the customer feedback.
• With coverage across 152 different product categories, the dataset provides a comprehensive sample that reflects the varied consumer behavior and preferences in Bangladesh's e-commerce landscape. This breadth of data is crucial for a holistic understanding of the market dynamics and customer sentiments in the region [1].
• Beyond its immediate utility for Daraz and Pickaboo, the dataset acknowledges the dynamic nature of e-commerce in Bangladesh, setting the stage for future expansions to include other e-commerce platforms. This potential for extension enhances the dataset's relevance and applicability in a rapidly evolving retail environment [2,3].
• The dataset's meticulously annotated reviews, categorized into five emotion types (Happiness, Sadness, Fear, Anger, and Love) and two sentiment classes (Positive and Negative), are invaluable for sentiment and emotion classification studies in NLP [4], [5], [6]. This detailed annotation enables precise analysis of customer emotions and sentiments, offering vital insights for businesses looking to optimize their product offerings and strategies.
• Serving as a pivotal resource for NLP research, the dataset enables the development of culturally and linguistically refined models that are critical for sentiment analysis. Its rich data composition also opens up possibilities for comparative analyses across various e-commerce platforms in Bangladesh, offering researchers and businesses alike the opportunity to delve into the intricacies of consumer behavior and preferences in different retail contexts [7,8].

## 2 Background

The drive for developing this dataset stemmed from a recognized need for a thorough and annotated compendium of product reviews specific to the Bangladeshi market [8]. In the dynamic realm of e-commerce, where platforms like Daraz and Pickaboo play a significant role amidst other popular platform [10], there was a notable gap in the availability of comprehensive resources for sentiment analysis and emotion classification in the Bangla language. This dataset addresses this gap by providing a large and diverse collection of product reviews from Daraz and Pickaboo. It is annotated with five emotion labels - Sadness, Fear, Anger, Happiness, and Love - and bifurcated into two sentiment classes, Positive and Negative, the dataset encompasses 78,130 samples across 152 product categories. By focusing on these two platforms, the dataset offers valuable insights into consumer behavior on these sites, while also acknowledging the potential for future research to encompass a broader spectrum of e-commerce platforms for a more holistic understanding of the Bangladeshi market. The dataset is not only instrumental for sentiment and emotion analysis within the Natural Language Processing (NLP) domain but also serves as a foundational tool for developing more culturally and linguistically tailored NLP models. The inclusion of diverse product categories and the rich linguistic context of the reviews make this dataset a significant resource for both specific and comparative analyses in the rapidly evolving field of e-commerce in Bangladesh.

## 3 Data Description

The research dataset, derived from public reviews on the Bangladeshi e-commerce platforms Daraz and Pickaboo, encompasses 152 categories, reflecting the diverse range of products available on these platforms. This dataset provides a comprehensive insight into consumer behavior and preferences in the Bangladeshi online retail market.

Daraz, Bangladesh's most popular e-commerce website, was founded in 2012. It is renowned for selling a wide array of products including electronics, appliances, clothing, fashion accessories, jewelry, footwear, furniture, and gadgets. Daraz not only dominates the Bangladeshi market but also has a significant presence globally, with a ranking of 8th nationally and 3781st internationally. The website attracts approximately 11.5 million visits, with an average of 7.63 pages per visit [9]. Acquired by the Alibaba Group in May 2018, Daraz operates across several countries including Bangladesh, Pakistan, Nepal, Sri Lanka, and Myanmar. Various sources including magenest.com [10], bdtype.com [11], prothomblog.com [12], and topinbangladesh.com [13] recognize Daraz as the top e-commerce website in Bangladesh, with an estimated 10.09 million monthly visitors. Additionally, it holds the 3rd position in the top 100 online stores in Bangladesh in 2023 [14].

Pickaboo, established in 2016, has quickly become a trusted online shop in Bangladesh, particularly known for its range of mobiles, computers, perfumes, watches, and electronic accessories. The platform reports an annual revenue between $15 M and $25 M, with a national rank of 551 and a global rank of 117986. It attracts around 498.1 K visitors and an average of 2.86 pages per visit [15]. Magenest.com reports that Pickaboo has approximately 361.91 K monthly visitors. According to bdtype.com [11] and prothomblog.com [12], Pickaboo is ranked 9th and 4th, respectively, among the top websites in Bangladesh. Furthermore, in the 2023 research of the top 100 online stores, Pickaboo is ranked 7th [14].

In this work, we present a comprehensive analysis of the Bangladeshi e-commerce market, showcasing our dataset's extensive diversity with a total of 152 product categories and 78,130 samples. This contrasts significantly with other datasets in the field. For example, the dataset discussed in [18], BANGLABOOK, is a large-scale dataset of Bangla book reviews with 158,065 samples classified into three categories: positive, negative, and neutral, but it only focuses on Bangla book reviews. In comparison, the dataset in [19], which focuses on reviews from Daraz, contains only 7,905 reviews. Additionally, a dataset from Kaggle for Bangla E-commerce reviews comprises only 949 reviews. These comparisons highlight the broader scope and larger size of our dataset.

Visualizing the product count for each category presents a challenge due to the sheer volume of categories, leading to a format that is not easily readable. For instance, as shown in Table 1, the product categories range widely, from 'Tools, DIY & Outdoor' with 6650 products to categories like 'MacBook' with just one product. This vast range demonstrates the breadth of consumer goods available on the platforms Daraz and Pickaboo. Moreover, the dataset is distinguished by its multi-label features. In line with Shaver et al.'s model [6], which identifies primary emotion categories such as happiness, anger, fear, and sadness, each product review in our dataset is annotated with a single emotion label based on these categories. Additionally, each review is classified under two sentiment classes – Positive and Negative – showcasing the dataset's multi-class properties.

**Table 1:** Product count for each category.

No.	Product Category	Product Count
1	Tools, DIY & Outdoor	6650
2	Watches	5845
3	Stationery & Craft	2703
4	Clothing	2595
5	Fruits, Meat & Frozen	2327
**…**	**…**	**…**
148	Case & Cover	1
149	Action Cameras	1
150	Microphone	1
151	VivoBook	1
152	MacBook	1

In developing this dataset, we made a conscious decision to omit the 'Neutral' sentiment category, focusing instead on capturing distinct emotional states and clear sentiment polarities. This choice aligns with our objective to analyze strong emotional expressions and sentiments in Bangladeshi product reviews. Such an approach enhances the dataset's utility for sentiment analysis and emotion classification tasks within the realm of Natural Language Processing. This focused categorization helps in deriving more pronounced and actionable insights from the consumer feedback, which is particularly valuable in understanding the emotional underpinnings of customer responses in the dynamic e-commerce environment.

**Fig. 1:**
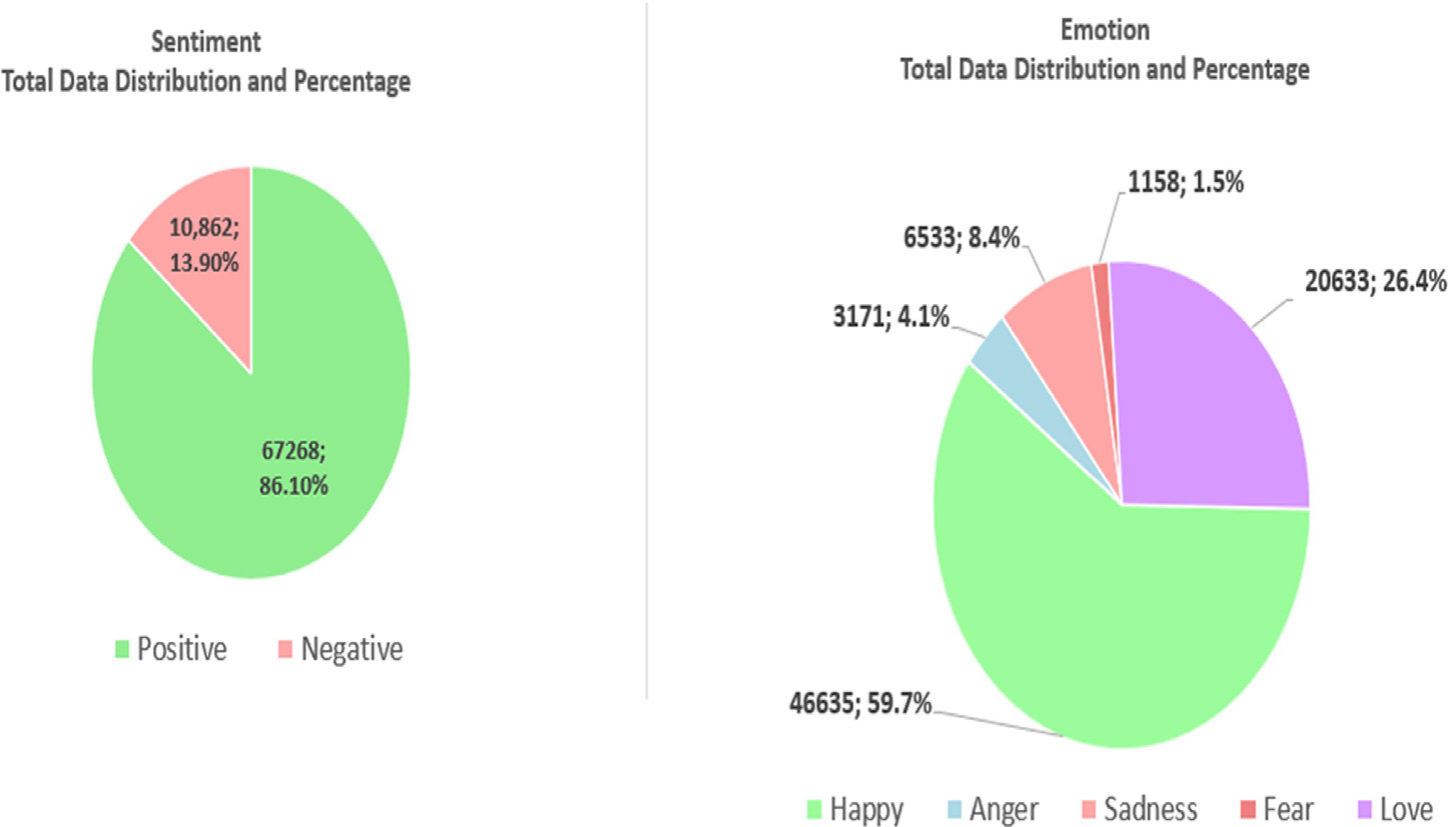
Distribution and percentage of sentiment and emotion data based on different classes.

This dataset comprises 78,130 samples from Bangladeshi e-commerce product reviews, split between 28,912 reviews in Bangla and 49,218 in English. Sentiment analysis reveals 10,862 instances of negative sentiment, making up 13.9 % of the total data. These negative sentiments are further classified into three emotion classes: Sadness (6,533 instances), Anger (3,171), and Fear (1,158). On the other hand, the dataset also includes a significantly larger portion of positive sentiment, with 67,268 instances, accounting for 86.1 % of the total data. This positive sentiment is divided into two emotion classes: Happiness (46,635 instances) and Love (20,633). After extensive pre-processing, the dataset presents a detailed distribution of data across these sentiment and emotion categories, as outlined in Table 2. Additionally, Fig. 1 provides a visual representation of this breakdown, further elucidating the distribution of emotions and sentiments within the dataset. These elements collectively underscore the comprehensive nature of the dataset and its nuanced approach to classifying emotions and sentiments in Bangladeshi e-commerce product reviews.

**Table 2:** Distribution of pre-processed data by sentiment and emotion categories.

Sentiment Class		Negative		Positive		
Emotion Class	Sadness	Anger	Fear	Happy	Love	
Total Data	6,533	3,171	1,158	46,635	20,633

The final output of our research is encapsulated in a CSV file named 'dataset.csv,' which is systematically structured to present our comprehensive findings. Table 3 representation an overview of sample data and column structure in the Dataset CSV. This file comprises seven distinct columns, each representing a specific aspect of the data collected:

• **Rating:** This column records the numerical rating given by the customer to the product, reflecting their overall satisfaction or dissatisfaction.
• **Review:** Contains the text of the customer's review, offering qualitative insights into their experience with the product.
• **Product Name:** Lists the name of the product being reviewed, providing context to the review and rating.
• **Product Category:** Indicates the category to which the product belongs, such as electronics, clothing, or appliances, helping in the categorization and analysis of data across different segments.
• **Emotion:** This column details the emotion attributed to the review, classified into categories like Happiness, Sadness, Fear, Anger, or Love, based on the sentiment expressed in the review.
• **Sentiment:** Identifies the overall sentiment of the review as either Positive or Negative, offering a quick summary of the customer's perspective.
• **Data Source:** Specifies the source of the review, for example, whether it was sourced from Daraz or Pickaboo, adding a layer of metadata that can be useful for further analysis.

**Table 3:** Overview of sample data and column structure in the dataset CSV.

## 4 Experimental Design, Materials and Methods

Three steps are used to prepare the dataset: data collection, data preparation, and exploratory analysis. The processes are briefly explained in this section.

### 4.1 Data Collection

**Fig. 2:**
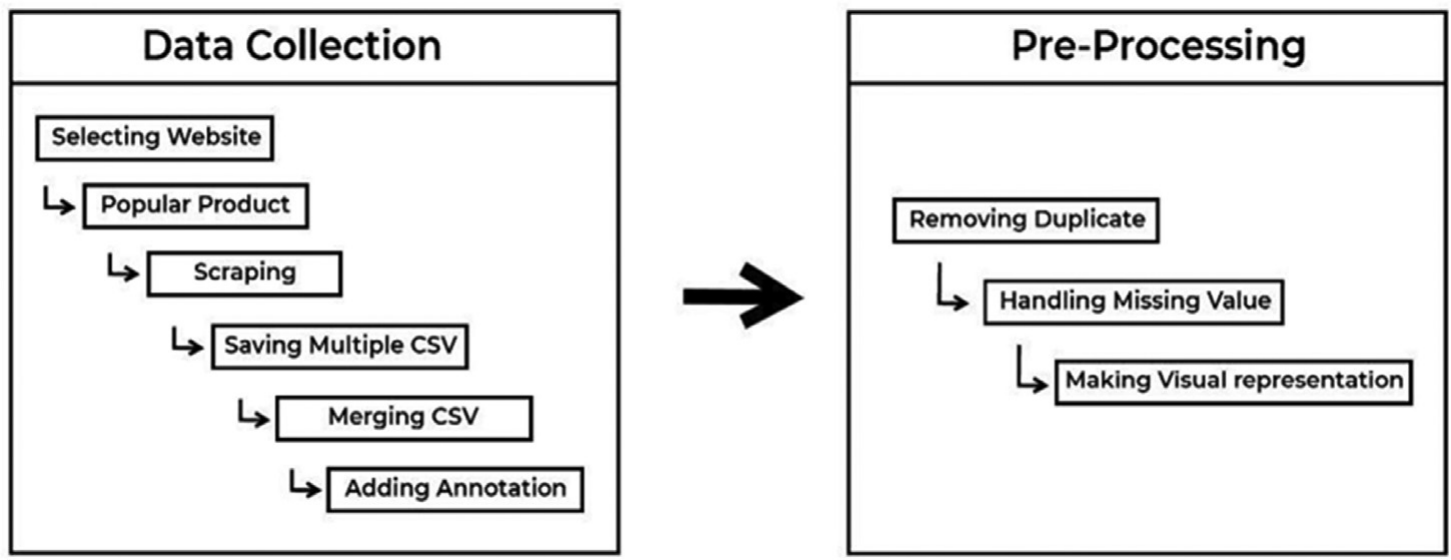
Flowchart illustrating the data collection and annotation process.

The data collection process for our research was methodically executed in several major steps, illustrated in a detailed flowchart in Fig. 2. The initial step involved selecting the e-commerce websites from which the data would be collected. Following this, we proceeded to the second step, which entailed choosing specific products from these websites for data scraping. The third step encompassed the actual scraping of reviews from the selected products. Once the reviews were collected, the subsequent phase involved the meticulous manual annotation of each review. This annotation process was crucial to accurately categorize each review with the relevant emotions and sentiments. The final step in our data collection methodology consisted of preprocessing tasks. These tasks included the crucial steps of removing duplicates and null entries, thereby ensuring the integrity and quality of the data in our dataset. This comprehensive process, from initial selection to preprocessing, is visually depicted in Fig. 2, providing an insightful overview of our methodical approach to data collection and annotation.

**Fig. 3:**
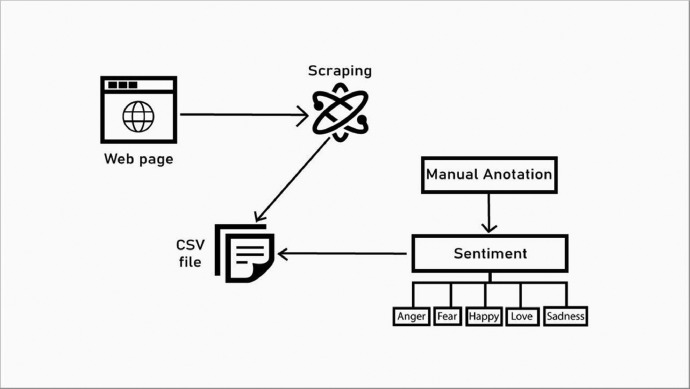
Web scrapping and data annotation process.
• **Data Annotation and Finalizing the Dataset:** The final step in our data collection was the manual addition of an 'emotion' column to the merged dataset. Each review was annotated based on Shaver's model [6], which includes five emotions: Anger, Love, Fear, Sadness, and Happiness. Reviews that did not distinctly express any of these emotions were initially labeled as 'neutral' but were later excluded from the final dataset to maintain a focus on clear emotional expressions.

Below is a detailed overview of the organized process:

• **Selection of E-commerce Platforms:** The first step involved choosing Daraz and Pickaboo, selected for their popularity and the extensive variety of products across diverse categories. These platforms are widely used by Bangladeshi online shoppers, providing a rich source of product reviews. This step was crucial in ensuring that our dataset reflected a broad spectrum of consumer opinions and captured the sentiments of the most interacted-with products.
• **Product Categories and Product Selection:** Given the impracticality of scraping reviews from all products due to time constraints and the extensive range of products on these platforms, our focus was on the most popular products, defined by the highest number of reviews. This selective approach aimed to maximize the representativeness and relevance of our dataset. We included all product categories available on these websites to ensure a comprehensive dataset that encompasses the most popular products in each category.
• **Web Scraping Process:** We employed Selenium in Python, operated on the Google Colab platform, for data scraping. For each selected product, its link was manually input into our code, generating a CSV file with details like rating, review, product name, and category for each product. After scraping the selected products, these individual CSV files were merged into a single file, streamlining the data for analysis and processing. Fig. 3 represents the meticulous approach to data collection, from the selection of platforms and products to the annotation and web scraping processes.

### 4.2 Data Preparation

For the annotation of emotions within our dataset, we strictly followed Shaver's model [6], a well-established framework in psychological research. Table 4 in our study delineates a hierarchy of emotions as outlined by this model, specifically tailored for the English language. This hierarchical structure is instrumental in guiding our process of accurately categorizing the emotional content present in the reviews. By employing this model, we were able to systematically classify each review into one of the predefined emotion categories: Anger, Love, Fear, Sadness, and Happiness.

**Table 4:** Hierarchy of emotions in English for sentiment analysis.

Emotion	Subordinate
Love	(1) adoration, affection, love, fondness, liking, attraction, caring, tenderness, compassion, sentimentality; (2) arousal, desire, lust, passion, infatuation; (3) longing
Happiness	(1) amusement, bliss, cheerfulness, gaiety, glee, jolliness, joviality, joy, delight, enjoyment, gladness, happiness, jubilation, elation, satisfaction, ecstasy, euphoria; (2) enthusiasm, zeal, zest, excitement, thrill, exhilaration; (3) contentment, pleasure, pride, triumph; (4) eagerness, hope, optimism; (5) enthrallment, rapture; (6) relief
Anger	(1) aggravation, irritation, agitation, annoyance, grouchiness, grumpiness; (2) exasperation, frustration; (3) anger, rage, outrage, fury, wrath, hostility, ferocity, bitterness, hate, loathing, scorn, spite, vengefulness, dislike, resentment; (4) disgust, revulsion, contempt; (5) envy, jealousy; (6) torment
Fear	(1) alarm, shock, fear, fright, horror, terror, panic, hysteria, mortification; (2) anxiety, nervousness, tenseness, uneasiness, apprehension, worry, distress, dread
Sadness	(1) agony, suffering, hurt, anguish; (2) depression, despair, hopelessness, gloom, glumness, sadness, unhappiness, grief, sorrow, woe, misery, melancholy; (3) dismay, disappointment, displeasure; (4) guilt, shame, regret, remorse, alienation, isolation, neglect, loneliness, rejection, homesickness, defeat, rejection, insecurity, embarrassment, humiliation, insult; (5) pity, sympathy

Our dataset includes a diverse linguistic mix, encompassing English text, Bangla text [7], and Bangla reviews written using English letters, along with reviews that consist solely of emojis. To address this variety, Table 5 presents the emotion hierarchy tailored for Bangla. This hierarchy is essential for accurately categorizing the emotional content in the Bangla reviews, ensuring that our sentiment analysis is comprehensive and culturally sensitive. Creating an emotion hierarchy tailored for Bangla within our dataset is motivated by the need for culturally and linguistically specific sentiment analysis tools [7,8]. Recognizing that emotional expression in Bangla may have unique nuances not fully captured by models based on other languages, we aimed to develop a framework that accurately reflects the emotional spectrum of Bangladeshi consumers. This endeavor ensures that sentiment analysis conducted using our dataset is more aligned with the cultural and linguistic contexts of the Bangla language, thereby enhancing the precision and relevance of the research findings in this specific linguistic domain.

**Table 5:** Hierarchy of emotions in Bangla for sentiment classification.

In our dataset, which comprises reviews from two different websites, we added a 'Website' column to indicate the source of each review. Additionally, a 'Sentiment' column was included, categorizing the emotional tone of each review as either positive or negative. Positive sentiment was assigned to reviews with 'Love' and 'Happy' emotions, while 'Anger,' 'Sadness,' and 'Fear' were marked as negative sentiments. This classification was based on the emotional content of each review, ensuring accurate sentiment analysis.

In our research, ensuring the reliability of manual data annotation was a critical aspect. To assess the agreement level between annotators, we employed the Cohen Kappa analysis. We first prepared a representative sample dataset from our main dataset, ensuring it encompassed all types of emotions and sentiments identified in our study. For the purpose of annotation, a new column was created for each annotator to record their agreement or disagreement on each item. We adopted a binary system where '+1′ indicated agreement and '-1′ indicated disagreement. The Cohen Kappa analysis results were interpreted using the scale proposed by Landis and Koch, which classifies agreement levels as follows: Less than 0.00 (Poor), 0.00 – 0.20 (Slight), 0.21 – 0.40 (Fair), 0.41 – 0.60 (Moderate), 0.61 – 0.80 (Substantial), and 0.81 – 1.00 (Almost Perfect).

Our analysis yielded the following results:

• Between Annotator 1 and Annotator 2, Cohen's Kappa score was approximately 0.44, indicating a moderate level of agreement.
• Between Annotator 1 and Annotator 3, the score was approximately 0.59, also suggesting a moderate level of agreement.
• The agreement between Annotator 1 and Annotator 4 was higher, with a Cohen's Kappa score of approximately 0.61, indicating a substantial level of agreement.
• Annotator 2 and Annotator 3 demonstrated an almost perfect level of agreement, with a score of approximately 0.90.
• The agreement between Annotator 2 and Annotator 4 was moderate, evidenced by a score of approximately 0.52.
• Lastly, Annotator 3 and Annotator 4 agreement was substantial, with a score of approximately 0.67.

On average, the Cohen's Kappa across all pairs of annotators was approximately 0.62, indicating a substantial level of agreement overall. This substantial agreement level amongst annotators reinforces the reliability of our manual annotation process, thereby enhancing the validity of our sentiment and emotion analysis in this study.

### 4.3 Exploratory Analysis

In our research, we faced a significant challenge in analyzing the extensive variety of product categories. We confronted the challenge of navigating through an expansive set of 152 product categories within our dataset. To enhance the efficacy of our study and ensure a focused examination, our exploratory analysis centered on the top-10 product categories. This selection was guided by a multi-faceted approach, considering review counts, ratings, and sentiments.

**Fig. 4:**
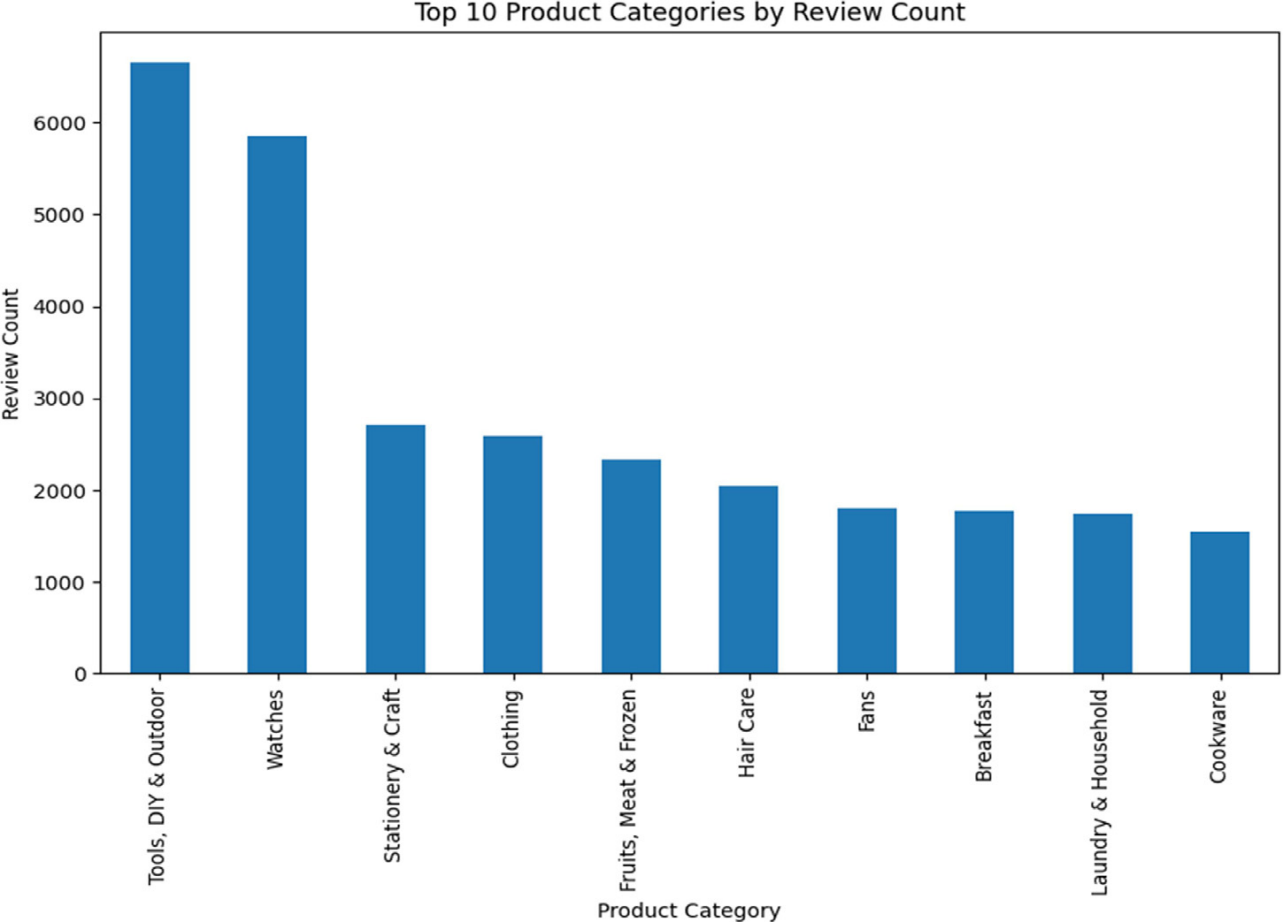
Product category Vs review count.

To determine the most relevant categories for our analysis, we initially conducted an assessment based on review counts. As depicted in Fig. 4, we identified the top-10 categories that garnered the highest number of reviews. It was observed that 'Tools, DIY & Outdoor' and 'Watches' received the most reviews. The subsequent four categories had review counts ranging from approximately 3000 to 2000, while the remaining categories received fewer than 2000 reviews each.

**Fig. 5:**
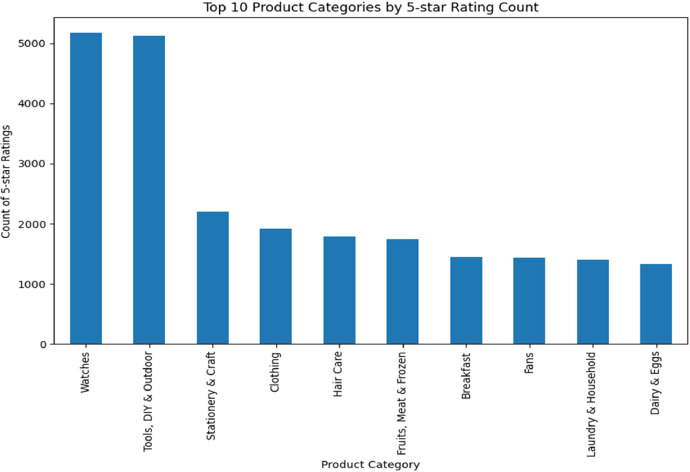
Product category Vs 5-star rating.

Recognizing the importance of product ratings in consumer behavior, we also analyzed the top-10 categories based on the count of the highest rating, which is 5.00. This analysis, shown in Fig. 5, revealed that the categories of 'Watches' and 'Tools, DIY & Outdoor' again emerged as leaders in receiving the most 5-star ratings. The next category in line received about 2100 5-star ratings, while the rest had fewer than 2000.

**Fig. 6:**
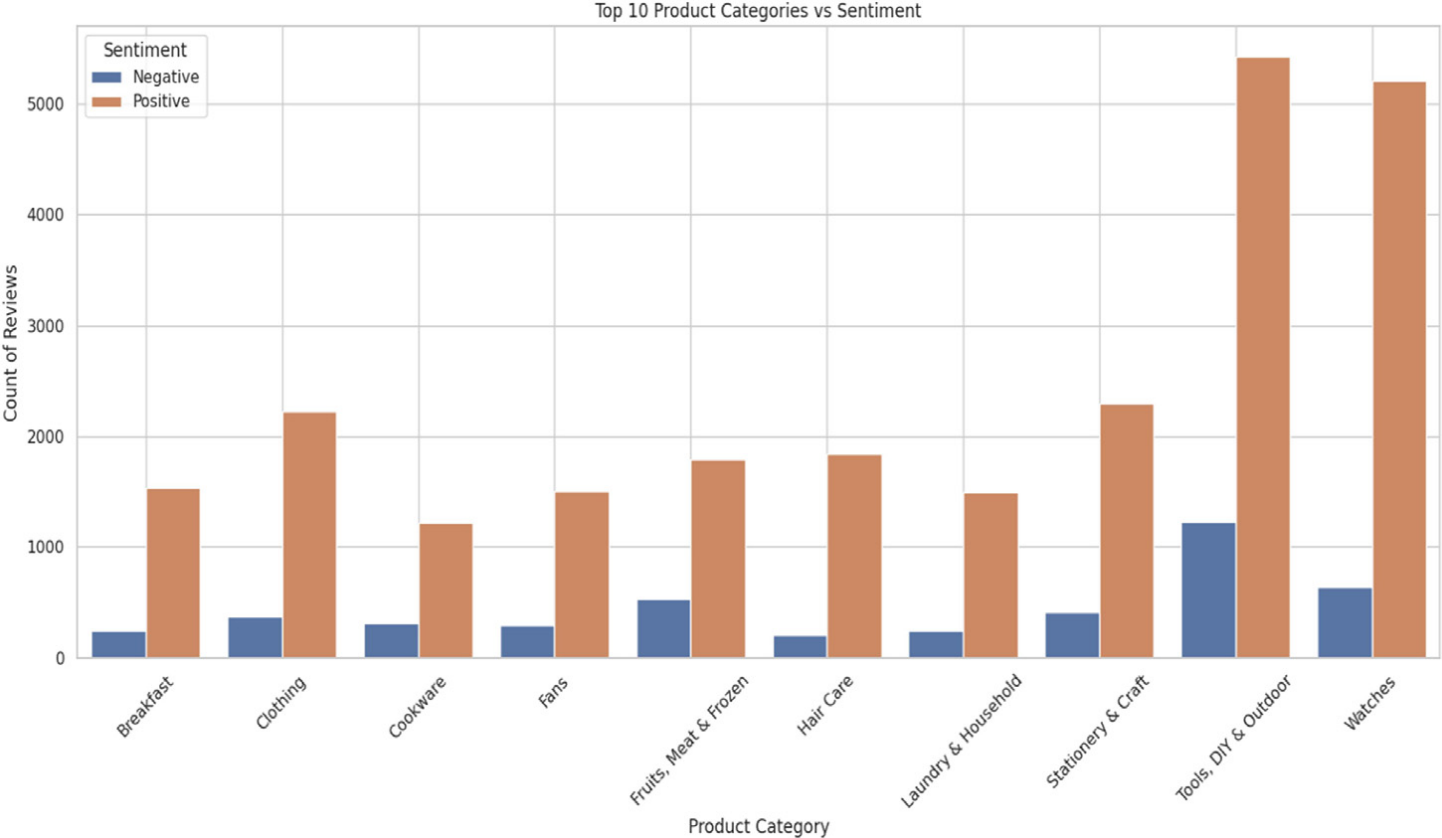
Product category vs sentiments.

Sentiment analysis was another critical aspect of our study. Using Table 1 as a reference, we knew our dataset contained both positive and negative sentiments, with positive encompassing 'Happy' and 'Love' emotions. As depicted in Fig. 6, we analyzed the top-10 categories based on the count of positive and negative sentiments. Again, 'Tools, DIY & Outdoor' and 'Watches' led in terms of positive sentiment, followed by two categories with nearly 2000 positive sentiments, and the remaining six categories had less than 2000.

**Fig. 7:**
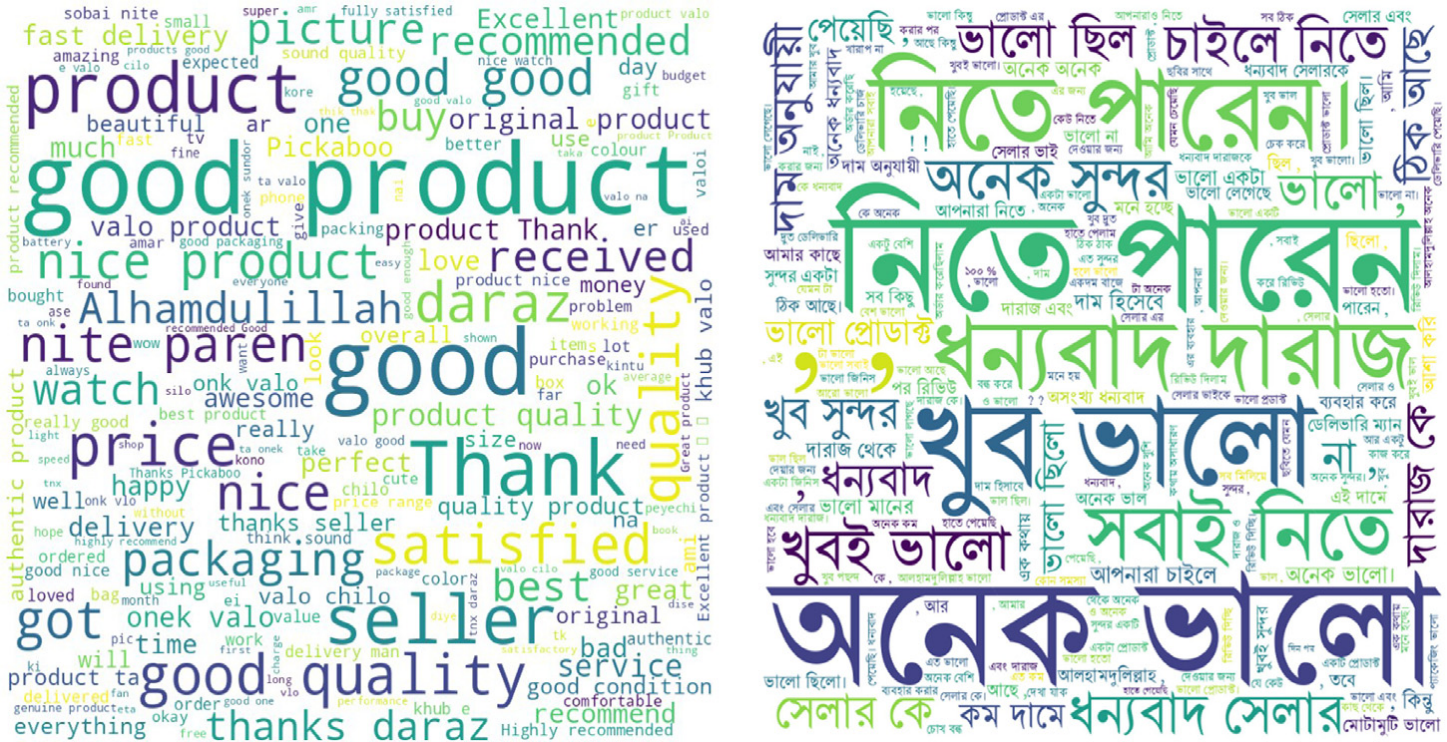
Word cloud for English and Bangla reviews.

Additionally, we created a word cloud (Fig. 7) to visualize the frequency of words in English and Bangla reviews across these categories. Through these focused analyses, we aimed to provide a clearer understanding of consumer preferences and behaviors within the most engaged product categories in the Bangladeshi e-commerce market.

## Limitations

The dataset under consideration does not encompass all product names. It primarily focuses on popular items from Daraz and Pickaboo, thereby did not take other products available on these platforms. Furthermore, the dataset has limited data sources, as it only includes data from these websites. Therefore, while this dataset offers valuable information on popular products from these websites, it may not provide a complete picture of the broader e-commerce landscape.

## Ethics Statement

All data in this study was collected by the authors. The dataset, derived from publicly available Bangladeshi e-commerce reviews, has been fully anonymized, with sensitive information like user IDs and names redacted to ensure confidentiality and compliance with privacy standards. This research adheres to ethical guidelines [16] and respects data redistribution policies [17]. No human subjects or animals were involved, and the data, used solely for research purposes and not for profit, does not include any content from social media platforms. The authors confirm adherence to the ethical standards of Data in Brief.

## CRediT authorship contribution statement

**Mohammad Rifat Ahmmad Rashid:** Supervision, Writing – review & editing, Conceptualization. **Kazi Ferdous Hasan:** Methodology, Software, Formal analysis, Data curation, Validation. **Rakibul Hasan:** Data curation, Investigation, Writing – original draft, Resources. **Aritra Das:** Project administration, Writing – original draft, Writing – review & editing, Data curation. **Mithila Sultana:** Visualization, Data curation, Writing – original draft, Writing – review & editing. **Mahamudul Hasan:** Supervision, Funding acquisition, Validation.

## Data Availability

A Multilabel Multiclass Sentiment and Emotion Dataset from Bangladeshi E-Commerce Reviews (Original data) (Mendeley Data)

## Footnotes

1 https://www.daraz.com.bd/

2 https://www.pickaboo.com/
